# A multi-institutional study of post-traumatic stress disorder and its risk factors in Ethiopian pediatric patients with physical trauma

**DOI:** 10.1186/s12888-022-03930-2

**Published:** 2022-04-15

**Authors:** Tadesse Tarik Tamir, Selam Fisiha Kassa, Daniel Ayelegne Gebeyehu

**Affiliations:** 1 Department of Pediatrics and Child Health Nursing, School of Nursing, College of Medicine and Health Sciences, University of Gondar, Gondar, Ethiopia; 2 Department of Community Health Nursing, School of Nursing, College of Medicine and Health Sciences, University of Gondar, Gondar, Ethiopia

**Keywords:** Pediatric, Post-Traumatic Stress Disorder, Trauma

## Abstract

**Introduction:**

Post Traumatic Stress Disorder (PTSD) was more common in children who had suffered physical trauma than in adults. Despite its prevalence, the prevalence and factors associated with PTSD in pediatric patients with physical trauma are unknown in Ethiopia. As a result, the purpose of this study was to determine the prevalence of PTSD and associated factors among pediatric patients with physical trauma who attended Northwest Amhara referral hospitals.

**Methods:**

An institutional-based cross-sectional study design was used in 422 pediatric patients with physical trauma aged 8–18 years from March 15 to May 15/2021. Using a systematic random sampling technique, data were collected from a sample of selected trauma patients via interviews and chart review. A standardized, pre-tested Child PTSD Symptom Scale was used to assess the severity of PTSD. Epidata 4.6 was used to enter the data, and Stata 14.0 was used to analyze it. Bivariable and multivariable binary logistic regression models were used to identify PTSD determinants.

**Result:**

The study included 422 paediatric patients who had suffered physical trauma, with a response rate of 97.87 percent. PTSD was found in 22.03 percent of paediatric patients with physical trauma in Northwest Amhara referral hospitals. The study discovered that female gender (AOR = 3.04, 95 percent CI: 1.58–5.84), age of 8 to 10 years old (AOR = 3.70, 95 percent CI: 1.39–9.87), having a chronic medical illness (AOR = 5.99, 95 percent CI: 2.60–13.77), having severe pain (AOR = 3.17, 95 percent CI: 1.12–8.99), low social support (AOR = 8.97, 95 percent CI: 4.04–19 were associated with PTSD.

**Conclusion and recommendation:**

The prevalence of PTSD was found to be high among pediatric patients who had experienced physical trauma. Special attention should be given to female patients, aged 8 to 10 years old, who have a chronic illness, for those who complain of severe pain and engaging others to provide good social support systems, are strongly recommended to alleviate PTSD in this segment of population.

## Introduction

**Fig. 1:**
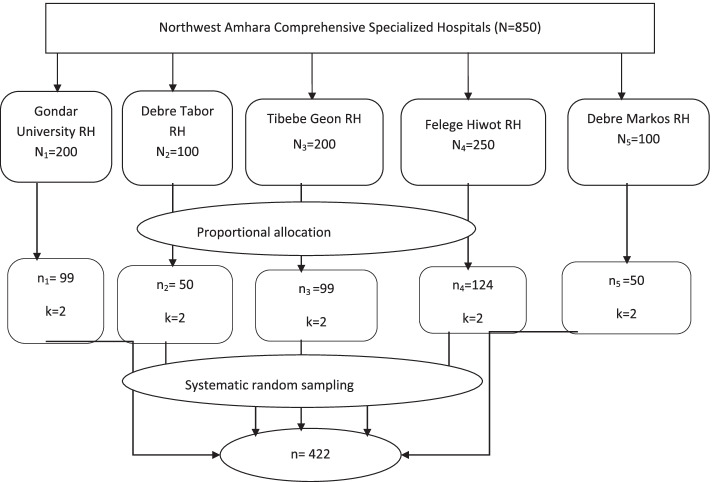
Schematic representation of sampling procedure for prevalence and associated factors of PTSD among pediatric patients with physical trauma in Northwest Amhara referral hospitals, 2021. *RH* Referral Hospital

PTSD is defined by exposure to a traumatic event, such as a severe motor vehicle accident, which is likely to result in recurrent, involuntary, and intrusive distressing memories of the traumatic event, as well as dissociative reactions [1]; moreover, the individual faced the mentioned traumatic event through either directly experiencing the traumatic event(s), witnessing, in person, the event(s) as it occurred to others, especially primary caregivers, and learning that the traumatic event(s) occurred to a parent or caregiving figure [1–3]. In studies, age, gender, address (place of residence), educational status, trauma cause, history of mental illness, family history of mental illness, and social support were associated with PTSD [4, 5].

Physical injury (trauma) is prevalent across many types of trauma experiences and can be associated with posttraumatic stress disorder (PTSD) symptoms [6]. It can be defined as a “body wound” caused by a sudden physical injury which mainly described as type 1 trauma [7].

According to a systematic review and meta-analysis of the prevalence of mental health conditions in ambulance personnel, the estimated prevalence of PTSD is around 11% [8]. A meta-analysis conducted in Australia revealed that the pooled prevalence of PTSD among children who had experienced physical trauma was 15.9% [9]. African studies reported a prevalence of PTSD among children ranging from 16% in Kenya to 55% in Uganda [10, 11].

Because the prevalence of PTSD following trauma has increased over time, it is critical to prioritise the disorder (PTSD) and develop strategies to address it in order to reduce the disorder's negative effects on health, particularly in young trauma survivors [12]. The proportion of people suffering from PTSD who seek hospitalization is low all over the world, but it is especially low in low to middle-income countries [13]. Because PTSD is a complex and chronic disorder that causes significant distress and interferes with social and educational functioning, improving PTSD detection may be an effective strategy [14]. Furthermore, the presence of PTSD in patients who have experienced physical trauma can have an impact on their clinical outcome and utilization of health resources [15]. It also has an effect on pediatric quality of life due to the consequences of functional and emotional impairment, and there is a negative cost to society with high financial and social consequences from significantly increased rates of hospitalization and suicide attempts [16]. Similarly, it was previously established that the economic impact of PTSD in a community is substantial [17].

Because the treatment options for a child who has experienced a physical trauma are primarily focused on restoring physical health, PTSD goes undiagnosed or untreated [18]. As a result, it will reduce quality of life, limit physical rehabilitation, and lengthen hospital stays in pediatric units; this is especially true in low-income countries [17]. Despite the fact that the risk of developing PTSD is higher in this segment of the population when compared to adults, no previous studies in Ethiopia demonstrated the magnitude and associated factors of PTSD among children with physical trauma. Therefore, understanding the problem could help local decision makers as it helps to design a comprehensive strategy to tackle the problem as early as possible. Furthermore, for clinicians, it will provide clues that early screening for PTSD and treating other related psychological problems for children admitted because of physical trauma (injury) will be mandatory. Likewise, it will help to provide an information to the family that is the caregiver on how they handle the emotional experiences of the child during the problem.

## Methods and materials

### Study area, design, and population

An institutional-based cross-sectional study was planned to run from March 15 to May 15, 2021. The study was conducted at Northwest Amhara Referral Hospitals. The Amhara regional state has a total population of 17,221,976 people, according to the Central Statistical Agency of Ethiopia in 2007, with 8,641,580 men and 8,580,396 women [19]. Currently, the Region has eight referral hospitals. Each Referral Hospital serves between 3.5 and 5 million people [20]. Five of the eight referral hospitals (Debre Markos, Felege Hiwot, Tibebe Gion, University of Gondar, and Deretabor) are located in Amhara's Northwest. The average flow of pediatric patients with physical trauma in University of Gondar, Debretabor, Tibebe Geon, Felege Hiwot, and Debre Markos referral hospitals in the three months preceding data collection was 200, 100, 200, 250, and 100 patients per month, respectively.

As a source population, all pediatric patients aged 8 to 18 years admitted to the orthopedics and surgical wards with physical trauma were used. Pediatric patients aged 8–18 years admitted to the orthopedics and surgical wards having a physical injury of 1 month and above duration who were present during the data collection period were considered the study population. All methods were carried out in accordance with ethical and human rights standards.

### Inclusion and exclusion criteria

The study included all pediatric patients aged 8 to 18 years admitted to surgical and orthopedic wards having a physical injury of at least 1 month duration. Individuals who have been seriously injured and are unable to respond properly to the questions, as well as those who have been diagnosed with PTSD prior to trauma, were excluded.

### Sample size and sampling procedure

The required sample size was estimated using a single population proportion formula by considering the following statistical assumptions; the prevalence of PTSD,50% (since there is no similar study conducted in the country among Paediatrics with physical injury [21], 95% confidence interval, 5% degree of precision, and 10% non-response rate, which yielded a total sample size of 422. The participant was selected every 2 intervals (K = 2) via the systematic sampling technique (Fig. 1).

### Data collection tools and procedures

The data was collected using standardized pretested questionnaires, interviewer-administered questionnaires. In addition, chart review was used to collect data regarding clinical factors. The questionnaire which was originally developed in English was translated into the local language (Amharic) by experts and translated back to English to check the consistency.. The questionnaire contains 52 questions divided into five sections: the first section includes six questions about the participants' socio-demographic characteristics, the second section includes four questions about clinical factors, the third section includes ten trauma-related questions, and the fourth section includes twelve questions about social support. The questionnaire regarding the trauma-related factors, clinical factors, and socio-demographic characteristics was adapted from different works of literature [22, 23].

Post-traumatic stress disorder (PTSD) was assessed using 20 items from the CPSS-SR-5, which was developed by the International Society for Traumatic Stress Studies (ISTSS) [24]. The CPSS-SR-5 is a DSM-5-adjusted version of the Child PTSD Symptom Scale Interview (CPSS-I). The 20 PTSD symptom items are rated on a 5-point frequency and severity scale ranging from 0 (never) to 4 (6 or more times per week/severe). The CPSS-SR-5 has good test–retest reliability (r = 0.800) and excellent internal consistency for total symptom severity (Cronbach's alpha = 0.924). In sum, the CPSS-SR-5 is a valid and reliable self-report instrument for assessing DSM-5 PTSD diagnosis and severity for children and adolescents aged 8–18 years [24]. Thus, those who scored ≥ 31 will be considered as having PTSD [25].

To assess social support, the Multidimensional Scale of Perceived Social Support was used (MSPSS). MSPSS is a self-report measure consisting of 12 items rated on a seven-point Likert scale that assesses an individual's perceived level of social support from family, friends, and significant others [26]. Participants with mean scale scores ranging from 1 to 2.9, 3 to 5, and 5.1 to 7 could be classified as having low, moderate, or high social support, according to the Multidimensional Scale of Perceived Social Support (MSPSS) [26].

The 0-to-10 Numerical Rating Scale (NRS) was used to assess pain intensity in trauma patients [27].

Physical injury (Trauma): can be explained as trauma that causes **a **body wound produced by sudden physical injury from Road traffic accident, Bullet/blast and Blow/assault, and Fall &Crush by a heavy object [7].

Duration of trauma: Patients with trauma lasting greater than 1 month after exposure are used in the diagnosis of PTSD, which may manifest up to 6 months after the trauma [28].

### Data quality assurance

To assure the quality of the data, a pre-test was conducted prior to the data collection on 5% of study participants at Dessie Referral Hospital. The reliability test was performed for CPSS-SR questionnaires using Cronbach’s alpha and initially, the value was 0.96. Training was given to data collectors regarding the techniques of interviewing, as well as filling the checklist for two-hours.

Experts translated the questionnaire from English to Amharic and back again to ensure that the same meaning was conveyed. Two trained Psychiatry nurses were assigned to collect data from each hospital through a face-to-face interview, and two Psychiatry nursing professional supervisors were on-site during the data collection period, reviewing all completed checklists in the evening of each data collection day to isolate incomplete and incoherent data.

### Data Processing and analysis

The data were double-checked for completeness and consistency. The data was then coded and entered into Epidata 4.6 before being exported to Stata 14.0 for further analysis. Descriptive statistics were computed, and the results were presented in the form of tables and graphs. The variance inflation factor (VIF = 1.23) and Hosmer–Lemeshow (*p* = 0.776) were used to test for multicollinearity and model goodness of fit, respectively.

To identify independent predictors of PTSD, bivariable and multivariable binary logistic regression were used. To control for potential confounding factors, all variables associated with PTSD with a p-value less than 0.2 in the bivariable analysis were further examined using multivariable analysis. Variables with a p-value of less than 0.05 were declared to be associated to PTSD.

## Result

### Socio-demographic characteristics of pediatric patients with physical trauma

The study included 422 paediatric patients who had experienced physical trauma, with a 97.87% response rate. Males made up more than two-thirds of the participants in the study (68.04%). The median (IQR) age of the participants was 15(12–15). A little more than half of the participants (51.33%) were aged 15 and older. The vast majority of them (72.15%) identified as orthodox Christians (Table 1).

**Table 1:** Socio-demographic characteristics of pediatric trauma patients in Northwest Amhara referral hospitals 2021(*n* = 413)

Variables	Category	Frequency (n)	Percent (%)
Sex	Male	281	68.04
	Female	132	31.96
Age	8–10	46	11.14
	11–14	155	37.53
	15–18	212	51.33
Religion	Orthodox	298	72. 15
	Muslim	72	17.43
	Protestant	36	8.72
	Catholic	5	1.21
	Adventist	2	0.48
Address	Rural	194	46.97
	Urban	219	53.03
Education	Unable to write and read	44	10.65
	Primary school	266	64.41
	Secondary &above	103	24.94
Caregivers educational status	Unable to write and read	105	25.42
	Primary school	101	24.46
	Secondary school	82	19.85
	Preparatory school	41	9.93
	College and above	84	20.34
Care givers Job	Farmer	181	43.83
	Merchant	137	33.17
	Government employee	81	19.61
	NGO employee	10	2.42
	Daily labor	4	0.97

*NGO* Nongovernmental organization

### Clinical related characteristics of pediatric patients with physical trauma

In terms of a history of chronic illness, more than three-quarters (81.36 percent) of the participants had none (Table 2).

**Table 2:** Clinical related characteristics among pediatric trauma patients in Northwest Amhara referral hospitals 2021(*n* = 413)

Variables	Response in Frequency (n) and Percent (%)
	**Yes**
Hypertension	11(14.29%)
Diabetes Mellitus	15 (19.48%)
HIV/AIDS	22 (28.57%)
Cardiac	5 (6.49)
Tuberculosis	8 (10.39)
Cancer	12 (84.42)
Asthma	25 (32.47)
**History of mental illness**	17(4.12)
**Family history of mental illness**	30 (7.26)

*HIV/AIDS* Human Immune Deficiency Virus/Acquired Immune Deficiency Syndrome

### Trauma and related factors among pediatric patients with/having physical trauma

More than half (53.75%) of the participants had experienced trauma to their lower extremities. A road traffic accident was nearly one-third (35.11%) of the participants' cause of injury. A large proportion (63.68 percent) of the participants suffered a fracture as a result of the accident. A significant majority (275(66.59%)) of study participants stated that the trauma occurred less than three months ago (Table 3).

**Table 3:** Trauma and related factors among pediatric patients with physical trauma in Northwest Amhara referral hospitals 2021(*n* = 413)

Variable	Category	Frequency (n)	Percent (%)
Site of trauma	Upper Extremity	125	30.27
	Lower Extremity	222	53.75
	Upper and Lower Extremity	66	15.98
Cause of trauma	Road traffic accident	145	35.11
	Bullet/blast and Blow/assault	140	33.90
	Fall &Crush by a heavy object	128	30.99
Type of trauma	Fractures	263	63.68
	Fracture and dislocation/sprain	112	27.12
	Ligament injury	30	7.26
	Blunt injury	5	1.21
	traumatic brain injury	3	0.73
Trauma related complications^a^	complications	275	66.59
	No complications	138	33.41
Duration of trauma	1–3 month	367	88.86
	3–6 month	46	11.14
Presence of amputation	Yes	21	5.08
	No	392	94.92
Pain	Yes	393	95.16
	No	20	4.84
Pain intensity(*n*^a^ = 393)	Mild	73	18.62
	Moderate	142	36.23
	Severe	177	45.15

Trauma related Complications: Gangrene, Amputation, and Infection

### Social support of pediatric patients with physical trauma

Among the participants, 206 (49.88%) had high social support, 24.21% had moderate social support, and 25.91% had low social support.

### Prevalence of PTSD among pediatric patients with physical trauma

Approximately 22.03 percent (95 percent CI: 18.3, 26.3) of pediatric patients with physical trauma in Northwest Amhara referral hospitals had PTSD, according to 413 respondents.

### Factors associated with PTSD among pediatric patients with physical trauma in Northwest Amhara referral hospitals, 2021

As shown in Table 4, bivariate analyses were performed to investigate the factors associated with PTSD in pediatric patients who had experienced physical trauma. As a result, approximately twelve variables were found to be significantly associated with PTSD: gender, age, education, chronic medical illness, mental illness history, family history of mental illness, cause of trauma, complication of trauma, duration of trauma, presence of amputation, severity of pain, and social support. Finally, multivariable logistic regressions were conducted and six variables are significantly associated with PTSD; The odds of presenting PTSD among females were 3.04 (AOR = 3.04, 95% CI: 1.58–5.84) times higher as compared to male participants, those children in the age group of 8–10 were 3.70 (AOR = 3.70, 95% CI: 1.39–9.87) times more likely to develop PTSD as compared to those 15-18 years old, patients with having any chronic medical illness were nearly 6 times more likely to be positive for PTSD as compared to their counterparts(AOR = 5.99, 95% CI:2.60–13.77), patients complaining a severe pain were 3.17 times more likely to develop PTSD as compared to those with mild pain (AOR = 3.17, 95% CI:1.12–8.99), those who had low social support were 8.97 times more likely to develop PTSD compared to high social support (AOR = 8.97, 95% CI:4.04–19.91) and respondents having moderate social support were 3.75 times more likely to develop PTSD as compared to having high social support (AOR = 3.75, 95% CI: 1.64–8.59) (Table 4).

**Table 4:** Binary logistic regression of factors associated with PTSD among patients pediatric with physical trauma in Northwest Amhara referral hospitals 2021(*n* = 413)

**Variables**	**Response**	**PTSD**		**COR(95% CI)**	**AOR (95% C**I)	***P* value**
		**Yes**	**No**			
Gender	Male	49	232	1.0	1.0	
	Female	42	90	2.21(1.37–3.57)	3.04(1.58–5.84)*	0.001
Age in year	8–10	11	30	2.22(1.11- 4.46)	3.70(1.39–9.87)*	0.009
	11–14	34	121	1.17(0.70–1.95)	1.72(0.82–3.64)	0.154
	15–18	41	171	1.0	1.0	
Educational status	Unable to write & read	12	32	5.06(1.04–24.63)	0.61(0.172–2.13)	0.434
	Elementary school	60	206	1.28(0.72–2.29)	0.82(0.36–1.84)	0.627
	Secondary & above	17	57	1.0	1.0	
Having Chronic medical illness	Yes	45	32	8.87(5.12–15.36)	5.99(2.60–13.77)*	0.0001
	No	46	290	1.0	1.0	
History of mental illness	Yes	8	9	3.35(1.25–8.96)	0.44(0.10–1.94)	0.280
	No	313	83	1.0	1.0	
Family history of mental illness	Yes	10	20	8.79(3.94–19.59)	3.17(0.96–10.50)	0.059
	No	312	71	1.0	1.0	
Cause of trauma	Road traffic accident	48	97	5.26(2.59–10.68)	2.31(0.96–5.62)	0.063
	Bullet and assault	32	108	3.15(1.51–6.56)	2.33(0.94–5.79)	0.067
	Fall down& crush by object	11	117	1.0	1.0	
Complication of trauma	Yes	74	201	2.62(1.48–4.65)	1.54(0.71 3.36)	0.274
	No	17	121	1.0	1.0	
Duration of trauma	1–3 month	74	293	0.43(0.22–0.83)	1.46(0.53–4.08)	0.465
	3–6 month	17	29	1.0	1.0	
Presence of amputation	Yes	12	9	5.28(2.15–12.98)	3.35(0.99–11.33)	0.052
	No	79	313	1.0	1.0	
Severity Pain(*n*^a^ = 393)	Mild	7	66	1.0	1.0	
	Moderate	22	120	1.73(0.70–4.26)	1.92(0.63–5.84)	0.248
	Severe	59	118	4.71(2.04–10.91)	3.17(1.12–8.99)^a^	0.030
Social Support	Low	48	59	10.36(5.41–19.82)	8.97(4.04–19.91)*	0.0001
	Moderate	28	72	4.95(2.50–9.81)	3.75 (1.64–8.59)*	0.002
	High	151	19	1.0	1.0	

## Discussion

The prevalence and associated factors of PTSD among pediatric patients with physical trauma in Northwest Amhara referral hospitals were investigated in this study. As a result, 22.03 percent of pediatrics with physical trauma were found to have PTSD, implying that it is a significant public health concern that necessitates a well-planned and comprehensive strategy to produce a healthy and fruitful generation. The prevalence of PTSD discovered in this study is consistent with a 22.5 percent prevalence found in a study conducted in the United States [29].

Nonetheless, it is significantly lower than studies conducted in Kenya (49%), Uganda (54.9%), and Palestine (54%), and slightly lower than India (30.6%) [11, 30–32]. This lack of clarity could be related to PTSD measurement tools (a study conducted in Kenya, Uganda, and Palestine used the Posttraumatic Stress Disorder Reaction Index (ULCA PTSD-RI), 16-item Harvard Trauma Questionnaire (HTQ-16), and Post-Traumatic Stress Disorders Symptoms Scale (PTSDSS), respectively) [11, 30–32]. The CPSS-SR-5, which has excellent reliability and good specificity, was used in this study, which could explain why the current study reported lower prevalence. Furthermore, male participants outnumber females in the current study. Despite the fact that boys in Ethiopia learn to suppress or deny their psychological symptoms than girls [33]. As a result, the child may have been prone to hiding his psychological symptoms; this could explain why the current study found lower prevalence.

On the other way, the finding was higher than studies in China 9.7%, Italy 1.9%, UK 9.6%, and Brazil 7.8% [34–37]. This discrepancy could be attributed to the study population; in this study, pediatric patients with physical trauma from any cause were included, rather than just single accident survivors, which could explain why the current study reported higher. This was also evidenced that prevalence of child PTSD after trauma differ with regard to the types of trauma included [38].

Regarding gender, being female has significantly increased the risk of PTSD. This is consistent with other studies done in Netherland and Korea [22, 39]. It is also consistent with a report of the WHO that also showed gender differences in the ability to cope with stress after a traumatic event [40]. Females are thought to be more sensitive to stress hormones and threats, with a lower likelihood of using effective coping strategies and a higher likelihood of negatively interpreting disasters than males, making them more prone to developing PTSD [41]. Furthermore, the DSM-5 identified gender (Female) as a risk factor for PTSD symptoms and diagnosis [30, 42]. In Ethiopia, females' traditionally burdened roles in society (such as increased household activity, early marriage against their will, and society's misperception of females being less important than males in economical contribution) may expose them to more stress. Early gender role socialization may also play a role, as boys learn to suppress or deny psychological symptoms, whereas girls learn to reflect their feelings more and become more emotionally expressive [33].

The study revealed that youngest children (8-10 years old) are more prone to develop PTSD than the middle (11–14 years old) and oldest age group (15–18 years old). This finding is supported by another studies[43, 44]. As evidenced by cognitive developmental models of PTSD [44], younger children are more vulnerable to developing PTSD than older children because they may have a poor protective mechanism to manage and interpret the traumatic event, and the difference in the development of both cognitive-affective regulation and getting peer group support. Nonetheless, these findings contradicted a study conducted in Palestine, which found that the middle age group was more vulnerable than the youngest and oldest ages [30]. It could be due to differences in lifestyle and environmental factors (like, socioeconomic resources, culture, availability and quality of health services, and etc.) influencing children's developmental stages across countries.

In the current study, pediatric patients diagnosed with chronic medical illness were more prone to develop PTSD. Previous studies have revealed similar result [45–48]. This could be explained by the fact that having a chronic illness could be a trigger event (stressor) for PTSD in paediatric patients, as having an accumulation of adverse conditions, such as illness, poly-medication, and a lack of a support network due to medical illness, increases the risk of PTSD [49–51].

The study found that experiencing pain with severe intensity was significantly associated with PTSD. This is consistent with previous studies [36, 52, 53]. It is possible that pain-related sensations can trigger the disorder's symptoms, like flashback (a somatic reminder of the traumatic experience), and re-experiencing symptoms [54].

Another important finding observed in this study is that pediatrics who had poor social support experienced PTSD. Other studies back up this finding [22, 55, 56]. Despite the fact that children with positive social support are less likely to develop PTSD as positive social support appears to mitigate the negative effects of traumatic injury [57]. Furthermore, low social support has also been linked to physiological and neuroendocrine markers of stress reactivity, such as increased heart rate, blood pressure, and exaggerated cardiovascular and neuroendocrine responses to stressors, making them more vulnerable to developing comorbid medical illness. [58, 59].

The study's strength was conducting the study in this understudied area of mental health (PTSD in Paediatrics), particularly in a country with a scarcity of research, which will help to design a comprehensive strategy to tackle the problem through early screening for PTSD and treating other related psychological problems in children. As the findings indicate, PTSD is more prevalent in this population segment, and it has been identified as an important public health concern that requires a well-designed comprehensive strategy. Furthermore, because injuries are a growing national concern in Ethiopia [60], the findings of this study will be used as a baseline data to design and implement preventive trauma-related mental health policies to reduce the psychological impact of the problem. Nonetheless, because PTSD can develop as a result of witnessing or learning about a traumatic event, it is critical to screen the child's parents or caregivers for the condition. Similarly, it would have been more novel if the study had been supplemented by qualitative studies to investigate possible childhood psycho-social development in relation to the country in which this study was conducted.

## Conclusion and recommendation

PTSD was found to be prevalent in paediatric patients who had experienced physical trauma. To alleviate PTSD in this population, special attention should be given to female patients, aged 8 to 10 years old, those who have a chronic illness and complain of severe pain. Furthermore, engaging others to provide good social support systems is highly recommended.

## Data Availability

All relevant data are available within the manuscript.

## Abbreviations

AIDS: Acquired Immune Deficiency Syndrome
AOR: Adjusted Odds Ratio
CI: Confidence Interval
COR: Crude odds ratio
CPSS-SR: Child PTSD Symptom Scale-Self Report
CSH: Comprehensive Specialized Hospital
DSM: Diagnostic and Statistical Manual
ISTSS: International Society for Traumatic Stress Studies
MSPSS: Multidimensional Scale of Perceived Social Support
NGO: Non-Governmental Organization
PTSD: Post Traumatic Stress Disorder
ULCA PTSD-RI: Posttraumatic Stress Disorder Reaction Index
WHO: World Health Organization
